# Reconciling carbon‐cycle processes from ecosystem to global scales

**DOI:** 10.1002/fee.2296

**Published:** 2021-02-01

**Authors:** Ashley P Ballantyne, Zhihua Liu, William RL Anderegg, Zicheng Yu, Paul Stoy, Ben Poulter, Joseph Vanderwall, Jennifer Watts, Kathy Kelsey, Jason Neff

**Affiliations:** ^1^ Department of Ecosystem and Conservation Sciences University of Montana Missoula MT; ^2^ Laboratoire des Sciences du Climat et de l’Environnement Gif-Sur-Yvette France; ^3^ CAS Key Laboratory of Forest Ecology and Management Institute of Applied Ecology Chinese Academy of Sciences Shenyang China; ^4^ School of Biological Sciences University of Utah Salt Lake City UT; ^5^ Department of Earth and Environmental Sciences Lehigh University Bethlehem PA; ^6^ Institute for Peat and Mire Research School of Geographical Sciences Northeast Normal University Changchun China; ^7^ Department of Biological Systems Engineering University of Wisconsin–Madison Madison WI; ^8^ National Aeronautics and Space Administration Goddard Space Flight Center Biospheric Sciences Laboratory Greenbelt MD; ^9^ Woods Hole Research Center Falmouth MA; ^10^ Geography and Environmental Science University of Colorado Denver Denver CO; ^11^ Sustainability Innovation Laboratory and Environmental Studies University of Colorado Boulder CO

## Abstract

Understanding carbon (C) dynamics from ecosystem to global scales remains a challenge. Although expansion of global carbon dioxide (CO_2_) observatories makes it possible to estimate C‐cycle processes from ecosystem to global scales, these estimates do not necessarily agree. At the continental US scale, only 5% of C fixed through photosynthesis remains as net ecosystem exchange (NEE), but ecosystem measurements indicate that only 2% of fixed C remains in grasslands, whereas as much as 30% remains in needleleaf forests. The wet and warm Southeast has the highest gross primary productivity and the relatively wet and cool Midwest has the highest NEE, indicating important spatial mismatches. Newly available satellite and atmospheric data can be combined in innovative ways to identify potential C loss pathways to reconcile these spatial mismatches. Independent datasets compiled from terrestrial and aquatic environments can now be combined to advance C‐cycle science across the land–water interface.

**In a nutshell:**

• From a societal perspective, there has never been a more urgent time to advance our understanding of the carbon (C) cycle, given that the atmospheric growth rate of carbon dioxide (CO_2_) has reached record levels
• From a scientific perspective, however, there has never been a better time to be a global ecologist, because global C observing systems are becoming more expansive and intensive, allowing scientists to make innovative insights at ecosystem, macrosystem, and global scales
• A fundamental goal of macrosystems research is to reconcile important processes from ecosystem to continental scales, which is now achievable using long‐term and consistent measurements of C‐cycle dynamics
• Comparisons across scales also reveal many CO_2_ loss pathways other than respiration that may not be included in ecosystem‐process models

Carbon (C) is the building block of life. Global photosyn‐thesis generates approximately 100 terawatts (TW) of energy each year by converting solar radiation into stored chemical energy (Barber 2009). Photosynthesis also represents the largest global annual C flux, of ~125 petagrams (Pg; where 1 Pg equals 10^15^ grams [g] and 1 Pg C is roughly equivalent to 0.47 parts per million [ppm] of CO_2_), with the second greatest flux consisting of the subsequent release of CO_2_ via respiration (~122 Pg C/year). Both of these fluxes are an order of magnitude greater than fossil‐fuel emissions (Ballantyne *et al*. 2015). The atmospheric CO_2_ that is fixed during photosynthesis is subsequently stored and transferred as chemical energy, which in turn fuels the metabolic reactions of most autotrophs and heterotrophs. Although C is the most common element in the terrestrial biosphere, representing approximately 50 parts per hundred (%) of all organic matter, CO_2_ represents only a very small fraction of the atmosphere and is therefore measured in ppm (~415 ppm in 2020). Given the abundance of C in the terrestrial biosphere and the massive fluxes of C occurring between the biosphere and the atmosphere, it is no surprise that scientists have developed a myriad of innovative ways for measuring and simulating C‐cycle processes across a range of scales in time and space. For example, chloroplast CO_2_ fluxes are estimated over millimeters per second, whereas biome CO_2_ fluxes may be estimated over thousands of kilometers per year. There have been many advances in C‐cycle science over the past 60 years at leaf, plant, ecosystem, and global scales, but both challenges to and opportunities for scientific advancement remain. Progress is necessary, however, especially at the macrosystem scale, where human management and ecological processes are often at odds and create interesting interactions of C dynamics.

One of the greatest impediments to accurate predictions of future climate is the uncertain response of the terrestrial C cycle to impending changes in temperature, precipitation, and atmospheric CO_2_ concentrations (Friedlingstein *et al*. 2013). Even though land‐surface models have become increasingly realistic in their mechanistic representation of C‐cycle processes by including nutrient limitation (Thornton *et al*. 2007), surface hydrology (Wang *et al*. 2013), and microbial processes (Wieder *et al*. 2013), this increased complexity does not necessarily reduce the range of uncertainty in projections of C uptake among models (eg see Friedlingstein *et al*. [2006] compared to Friedlingstein *et al*. [2013]). In parallel, there is now a globally nested CO_2_ observation network that allows for unprecedented measurements of changes in CO_2_ concentrations and fluxes (Schimel *et al*. 2015). These continuous measurements allow estimates to be made of net CO_2_ exchange from ecosystem to global scales, but not necessarily the underlying processes that regulate this net exchange (Ciais *et al*. 2019). In contrast, land‐surface models simulate the underlying processes that result in net CO_2_ exchange, but these are difficult to benchmark due to a lack of process‐level data at the appropriate scale (Luo *et al*. 2012; Anav *et al*. 2013).

Although enhanced net C accumulation in the terrestrial biosphere can be inferred from the global C budget, identifying the ecosystems in which C is accumulating is still difficult. For example, at the global scale, it can be concluded with confidence that ~25% of CO_2_ emitted to the atmosphere from fossil‐fuel and land‐use emissions has been taken up by the terrestrial biosphere (Ballantyne *et al*. 2015; Le Quéré *et al*. 2016), but biomass datasets are too sparse in extent or too short in duration to document which ecosystems continue to accumulate C. More detailed ocean and land measurements now make it possible to identify specific processes affecting the net CO_2_ atmospheric exchange between the marine biosphere (Landschützer *et al*. 2015) and terrestrial biosphere (Anderegg *et al*. 2015), in some instances at regional scales (Ciais *et al*. 2019). However, partitioning net C fluxes into their component gross fluxes of photosynthesis and respiration remains a challenge (Wehr *et al*. 2016).

Another vexing problem in global C‐cycle research is that top‐down global estimates of net terrestrial C uptake do not agree with bottom‐up ecosystem estimates when integrated globally. For instance, top‐down estimates of global net terrestrial C uptake in 2010 are an order of magnitude less (2.2 ± 2.1 Pg C/year; Ballantyne *et al*. 2015) than eddy covariance estimates up‐scaled globally (22 ± 5 Pg C/year; Jung *et al*. 2011). Although some of the discrepancy between top‐down and bottom‐up estimates of net terrestrial C uptake may be due to issues associated with eddy covariance methods (Keenan *et al*. 2019) – particularly regarding measurement of nighttime respiration, which often violates eddy covariance requirements of turbulent flux and biases in the sampling network – a portion can also be explained by non‐respiratory CO_2_ loss pathways (~7 Pg C/year; Randerson *et al*. 2002). This suggests that there are many C transformation and transport pathways that ultimately lead to a loss of CO_2_ from ecosystems back to the atmosphere. Approximately 90% of inland lakes and streams are net sources of CO_2_ to the atmosphere (Cole *et al*. 1994), and at a global scale approximately 2 Pg C/year is returned to the atmosphere via CO_2_ loss from rivers and lakes (Raymond *et al*. 2013). Although this estimate of CO_2_ loss from aquatic ecosystems is comparable to the magnitude of net C uptake by terrestrial ecosystems, it is less than 2% of total inferred CO_2_ respiration from the terrestrial biosphere back to the atmosphere (Ballantyne *et al*. 2017). As such, characterizing the C balance at the macrosystem scale for direct comparison with different biomes in Earth system models remains difficult (Peylin *et al*. 2013).

Although from a societal perspective there has never been a more urgent time to study the C cycle and its sensitivity to climate change (Obama 2017), from a scientific perspective there has never been a more exciting time to study C‐cycle processes. The global C observation network supports innovative analyses and syntheses across scales from ecosystems to the entire planet. Currently, there are over 800 eddy covariance sites operating around the world that contribute measurements of net CO_2_ exchange, as well as estimates of primary productivity and total respiration across a wide array of ecosystems (Chu *et al*. 2017). However, in the US, fewer than half of the ecosystem functional types are represented in the combined core sites of the AmeriFlux Network and the National Ecological Observatory Network (NEON) (Villarreal *et al*. 2018), and many ecosystems remain underrepresented, especially in climate‐sensitive Arctic tundra and tropical rainforests. Other C flux databases have continued to expand, such as a recently updated database on soil respiration that has been used to identify the climate sensitivity of soil respiration over time (Bond‐Lamberty and Thomson 2010), which is critical for evaluating how C supply, soil temperature, and moisture interact to regulate soil respiration (Hursh *et al*. 2017).

Global measurement networks and satellite observations of atmospheric CO_2_ now allow for the characterization of biome‐scale C fluxes at greater temporal and spatial resolutions (Figure 1). The global greenhouse observation network has grown sporadically, with approximately 90 in situ sites now in operation worldwide (GLOBALVIEW‐CO_2_ 1999). Several of these sites also provide atmospheric profile measurements that are essential for estimating latitudinal differences in CO_2_ exchange (Stephens *et al*. 2007), in addition to seasonal differences in regional uptake (Gatti *et al*. 2014). Regional atmospheric CO_2_ monitoring networks often engage in intensive atmospheric campaigns to better define regional C fluxes in urban continental settings (Corbin *et al*. 2010) or to determine recent changes in the C balance of ecosystems in climate sensitive regions, such as the Arctic (Commane *et al*. 2017). When combined with three‐dimensional atmospheric transport modeling and estimates of surface fossil‐fuel emissions, these so‐called “atmospheric inversions” deliver critical information about the net exchange of CO_2_ at biome scales (Peylin *et al*. 2013). The array of Earth observing satellites has also grown tremendously, providing better spatiotemporal coverage of vegetation indices that are useful for assessing patterns and trends of global productivity since ~1982 (Pinzon and Tucker 2010), as well as valuable information on changes in vegetation cover (Song *et al*. 2018) and ecosystem stress (Anderegg *et al*. 2018). Recent advances in satellite observations facilitate quantification of concentration estimates integrated over the entire total atmospheric column for CO_2_ (ie XCO_2_) and CH_4_ (ie XCH_4_). Although potentially less precise than those relying on surface measurements using infrared gas analyzers, these estimates provide more continuous global coverage, improving characterization of regional flux anomalies and attribution to specific C‐cycle processes (Liu *et al*. 2017).

**Figure 1:**
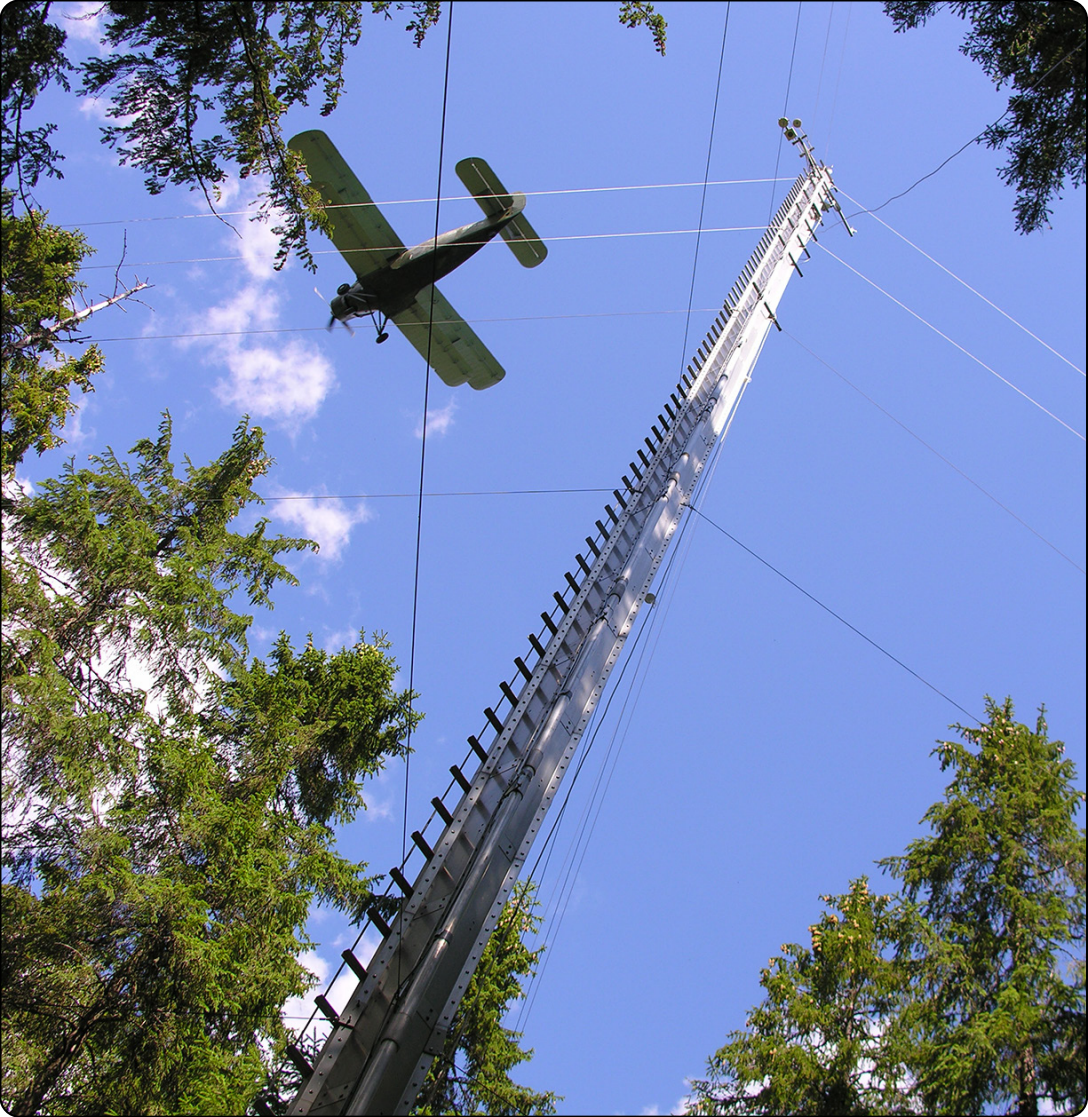
Image of airborne observations combined with eddy flux observations of carbon (C) fluxes to measure ecosystem–atmosphere exchanges of carbon dioxide (CO_2_). A Varlagin/imaggeo.egu.eu

Innovative ways to combine ecosystem measurements with satellite observations have made it possible to quantify how different ecosystems are responding to concomitant changes in atmospheric composition, including CO_2_ concentration, surface temperatures, and regional precipitation. Moreover, these top‐down and bottom‐up observations are helping researchers to disentangle net C exchange into its component processes of photosynthesis and respiration across various scales, which provides important diagnostics for models that are designed to simulate the concurrent ecological processes and not just net CO_2_ exchange. For instance, combined satellite and meteorological observations have been used in a machine‐learning framework to up‐scale eddy covariance measurements to provide spatially and temporally continuous estimates of global primary productivity (Jung *et al*. 2011). Likewise, global atmospheric CO_2_ measurements have been used to constrain net CO_2_ exchange in combination with satellite data to constrain primary productivity to infer the uncoupling of photosynthesis and respiration on decadal timescales (Ballantyne *et al*. 2017). The challenge for the scientific community is figuring out ways in which emergent patterns of net CO_2_ exchange can be used (Cox *et al*. 2013) to identify underlying mechanistic processes that can be diagnosed in models (Anderegg *et al*. 2015). Ultimately, this will lead to scientific advances and societal benefits through improved Earth system models with less uncertainty in future climate predictions.

## Theoretical representation of C‐cycle processes

**Figure 2:**
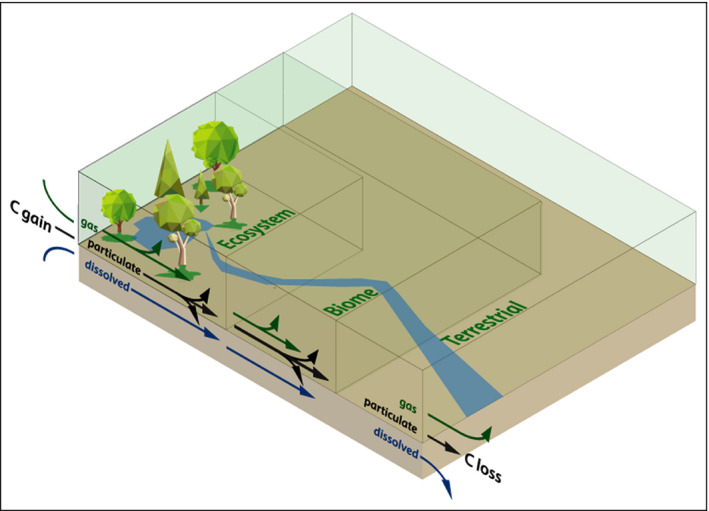
Conceptual figure showing pathways of C gain and loss from ecosystem to biome to terrestrial scales within the biosphere. Although it is often assumed that very little change occurs among the gas, particulate, and dissolved phases of C, ecosystems are very effective at transforming C, such that C gain pathways may not correspond with C loss pathways, leading to an apparent C imbalance across scales. Furthermore, C can be transported across scales via either advection through the atmosphere or fluvial processes in aquatic ecosystems.

Although the global C observing system has been greatly expanded and advanced over the past six decades, the theoretical and conceptual framework for understanding C‐cycle dynamics has not necessarily kept pace (Figure 2). There has been extensive discussion over the past several decades concerning how the biosphere–atmosphere C exchange can best be defined. The challenges in defining C exchange lie across several axes, including time, space, and C form. Additional issues arise from the different processes occurring in and the transfer of C between aquatic and terrestrial ecosystems. Although we focus solely on terrestrial processes occurring from the ecosystem to biome scale here, we acknowledge the importance of the aquatic interface (Butman *et al*. 2018). The evolution of C‐cycle measurements and key issues regarding terminology was described by Chapin *et al*. (2006), who defined net ecosystem C balance (NECB) simply as the change in C per unit time, but then broke this measure down into its component fluxes:

((Equation 1).) $$\text{NECB}=\text{NEE}-F_{\text{CO}}-F_{\text{VOC}}-F_{\text{CH}4}-F_{\text{DIC}}-F_{\text{DOC}}-F_{\text{PC}}$$

In this formulation, net ecosystem exchange (NEE) is a measure of the net ecosystem CO_2_ exchange as the difference between gross primary productivity (GPP) and total ecosystem respiration (TER), and at the ecosystem scale is typically measured using eddy covariance techniques (Wofsy *et al*. 1993). Although the CO_2_ flux associated with NEE is usually the dominant form of net C exchange in many ecosystems, it cannot be assumed that transformations of C do not occur as a result of ecosystem processes. For instance, fluxes of carbon monoxide (*F*_CO_), volatile organic compounds (*F*_VOC_), methane (*F*_CH4_), dissolved inorganic C (*F*_DIC_), dissolved organic C (*F*_DOC_), and particulate C (*F*_PC_) all represent C loss pathways that may affect the net C balance over time. Although NEE is sometimes used synonymously with NECB, it is an approximation that can, under certain circumstances, leave out quantitatively important non‐respiratory processes that contribute to ecosystem C balance.

A second key issue in C balance terminology emerges at larger spatial or temporal scales when other factors can become major contributors to C balance. Notably, large disturbances like wildfire, landslides, and insect infestations can cause large or punctuated redistributions of C. In human managed ecosystems, activities such as logging, harvest, and other forms of C transfer can result in C taken up at the ecosystem scale being lost at the biome scale, and this transfer can actually cause NECB to shift from a net C sink to a net C source. The net biome productivity (NBP) concept was first introduced by Schulze *et al*. (2000) to account for C transfer and subsequent loss at regional scales. In Chapin *et al*. (2006), NECB represents NBP integrated over fixed space and time domains, with the assumption that additional processes analogous to those shown in Equation (1) may need to be added to account for C fluxes driven by periodic events. At the global scale, we can assume that CO_2_ mass is conserved in the atmosphere and thus, given fossil‐fuel emissions to the atmosphere and estimates of net CO_2_ uptake by the oceans, net CO_2_ uptake by the terrestrial biosphere can be inferred (Le Quéré *et al*. 2016). More importantly, the atmosphere and oceans provide constraints on global C exchange because these are relatively well‐mixed homogenous reservoirs as compared to ecosystem C pools and fluxes that tend to be much more spatially and temporally heterogeneous. Resolving C‐cycle processes from ecosystem to global scales may therefore require an update to C‐cycle nomenclature (see WebPanel 1).

## Spatial scale differences in C balance

**Figure 3:**
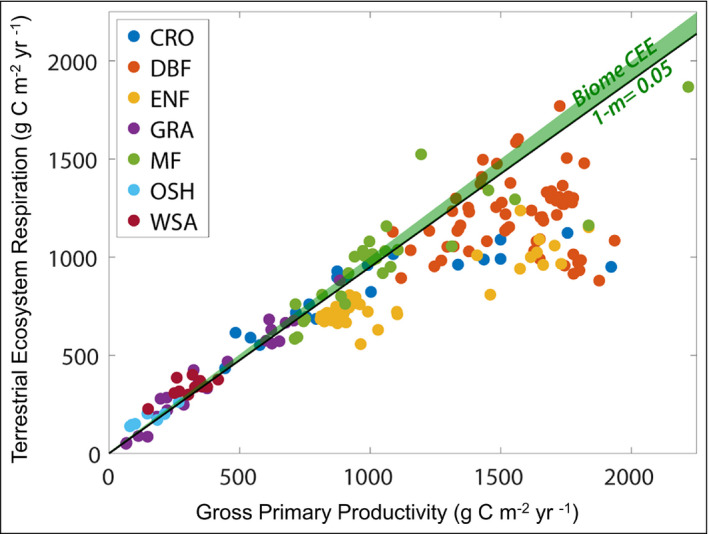
Comparison of C exchange efficiency (CEE) at ecosystem to biome scales across the continental US. Each point represents the mean annual gross ecosystem productivity and total ecosystem respiration for cropland (CRO), deciduous broadleaf forests (DBF), evergreen needleleaf (ENF), grassland (GRA), mixed forest (MF), open shrubland (OSH), and woody savanna (WSA) eddy covariance sites across the US. The diagonal line was derived from satellite estimates of gross primary productivity (GPP) and atmospheric estimates of net CO_2_ exchange at the scale of the continental US and indicates that 95% of C fixed during photosynthesis is lost to the atmosphere through respiration, or that CEE is only 5% (1 – 0.95 = 0.05), represented by the green wedge.

The “C exchange efficiency” (CEE = NEE/GPP) may provide a useful framework (see WebFigure 1) for comparing relative fluxes across ecosystem to global scales. At the global scale, only ~2% of C fixed annually through GPP remains in the biosphere as a result of NEE (2.5/125 Pg C/year), suggesting that CEE of the terrestrial biosphere is remarkably low. At the scale of the continental US, approximately 5% of C fixed annually through photosynthesis remains in the terrestrial biosphere (Figure 3). However, estimates of CEE derived from eddy covariance methods reveal very large differences among terrestrial ecosystems. Ecosystems with lower levels of GPP tend to fall on the CEE line at the continental scale, whereas more productive ecosystems tend to deviate from the CEE line. For example, grasslands have very low CEE (~2%), a level consistent with the global CEE, whereas evergreen needleleaf forests and deciduous broadleaf forests appear to have quite high CEE values (~31% and ~24%, respectively). Therefore, our ecosystem‐scale measurements suggest that these forests are strong C sinks, whereas our global‐scale measurements suggest that much of this apparent forest C uptake is lost, indicating that these forests may be acting more like “C sieves”. Moreover, croplands vary considerably, with less productive croplands falling on the continental CEE line and more productive croplands deviating considerably, with an overall CEE of 23%. It should be noted, however, that according to mass balance, the integral of NEE across all ecosystems (aquatic and terrestrial) should be equal to global NEE; in other words, CEE estimates from the different ecosystems shown in Figure 3 should all fall on the continental CEE line (Chapin *et al*. 2006). Therefore, measurements of net CO_2_ exchange at the ecosystem scale are biased, or CO_2_ loss pathways at the continental scale are offsetting the apparent net uptake of CO_2_ by certain ecosystems.

Measurement biases of ecosystem C fluxes stem from the location of eddy flux sites or systematic biases in NEE measurements. In the US, this network bias should be reduced with the addition of more observation sites, such as NEON sites; however, there remain notable gaps in the intermountain west, north‐central plains, and parts of the Southeast. Also noteworthy is that eddy flux sites are often situated in rapidly regenerating ecosystems and as such may not capture the full trajectory of ecosystem C dynamics (Luyssaert *et al*. 2008). Furthermore, the eddy flux approach only measures the net ecosystem CO_2_ exchange (ie NEE) directly, whereas photosynthetic fluxes and total ecosystem respiration fluxes are estimated, resulting in the potential for systematic biases to occur in these measurements. Eddy covariance methods are inherently challenging in ecosystems with dense canopies (Thomas *et al*. 2013), which can lead to nocturnal C storage within the canopy (Fu *et al*. 2018) and decoupling of the canopy and the atmosphere that may vary seasonally (Jocher *et al*. 2017). This may help explain the strong divergence between both deciduous broadleaf and evergreen needleleaf forests and the CEE line at the continental scale (Figure 3). If daytime respiration is reduced with respect to nighttime respiration, large overestimates of both photosynthetic gains and respiration losses at the ecosystem scale may result, which would increase relative ecosystem CEE (Keenan *et al*. 2019).

The discrepancy between CEE at the biome scale and the ecosystem scale can also be explained by the lack of measurements of non‐respiratory loss pathways of CO_2_ back to the atmosphere (Figure 2). For example, aquatic ecosystems, which are effective at transporting dissolved and particulate forms of inorganic and organic C and transforming it to CO_2_ such that it may be lost to the atmosphere (Neff and Asner 2001; Hotchkiss *et al*. 2015), were not plotted on our diagnostic CEE plot (Figure 3). There are many measurements of the partial pressure of CO_2_ in aquatic environments, which determine whether CO_2_ is diffusing in or out of aquatic ecosystems, but these are not always combined with productivity estimates (albeit see Hotchkiss *et al*. 2015; Bernhardt *et al*. 2018). Volatile organic C (VOC) compounds are another major source of C loss from ecosystems, which may help to reconcile the discrepancy between ecosystem‐ and biome‐scale C exchange efficiencies. Estimates of VOC production are tightly coupled to primary productivity and range around 450 teragrams (Tg) C/year, making them a very small fraction of terrestrial GPP (less than 0.4%) but an appreciable fraction of NEE (~15%), assuming that VOCs are rapidly oxidized to form CO_2_ (Unger *et al*. 2013). Finally, the only ecosystem‐scale C loss pathways that can help reconcile ecosystem‐ and global‐scale estimates of CEE are oxidative pathways that ultimately lead to atmospheric CO_2_ (eg CO_2_ emissions from wildfires), meaning that other loss pathways leading to reduced C (eg CH_4_ emissions) will not help reconcile these discrepancies of scale.

**Figure 4:**
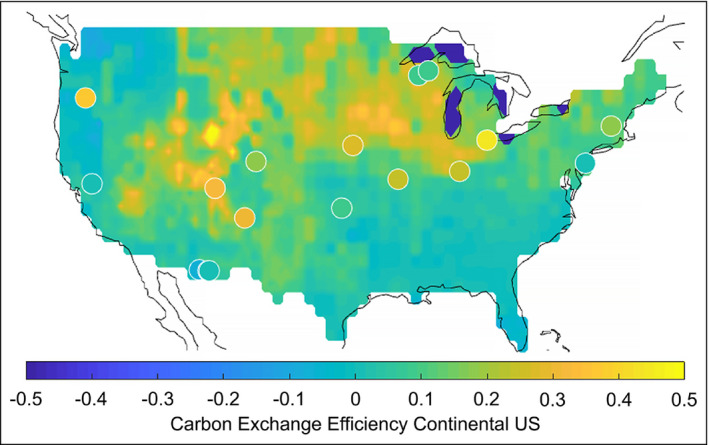
CEE for the continental US. Spatially continuous estimates of GPP derived from Moderate Resolution Imaging Spectroradiometer (MODIS) satellite estimates and continuous estimates of net ecosystem exchange derived from atmospheric inversions over the continental US (Peters *et al*. 2007) are compared with in situ ecosystem‐scale measurements made at 16 different eddy covariance core sites within the AmeriFlux Network (circle points). Positive values indicate regions where ecosystems are a net sink of C from the atmosphere, whereas negative values indicate regions where ecosystems are a net source of C to the atmosphere.

We can also look at the spatial distribution of CEE from the biome to ecosystem scale (Figure 4). At the continental scale in the US, it is apparent that high CEE in the midwestern region near the Great Lakes is driven primarily by high mean annual NEE, and very high CEE in the intermountain west is driven by low GPP and modest NEE. In contrast, highly productive regions, such as the Pacific Northwest and the Southeast, do not necessarily retain a large fraction of GPP as NEE, as reflected in their relatively low CEE values. These regional differences in CEE seem to be corroborated by eddy covariance sites in certain biomes, such as the Northeast and parts of the Southwest, but less so in other regions. There appears to be a strong mismatch in CEE near the Great Lakes, with regional estimates suggesting a relatively high CEE, whereas eddy flux sites indicate a much lower CEE. This may be due to the specific locations of eddy flux sites that may not capture the diverse array of midwestern ecosystems. A similar mismatch is evident in the Pacific Northwest, where regional CEE values are extremely low – and in some instances negative – due to an apparent net source of CO_2_ to the atmosphere, while eddy flux measurements from central Oregon suggest high CEE.

## Climate sensitivity of C‐cycle processes

**Figure 5:**
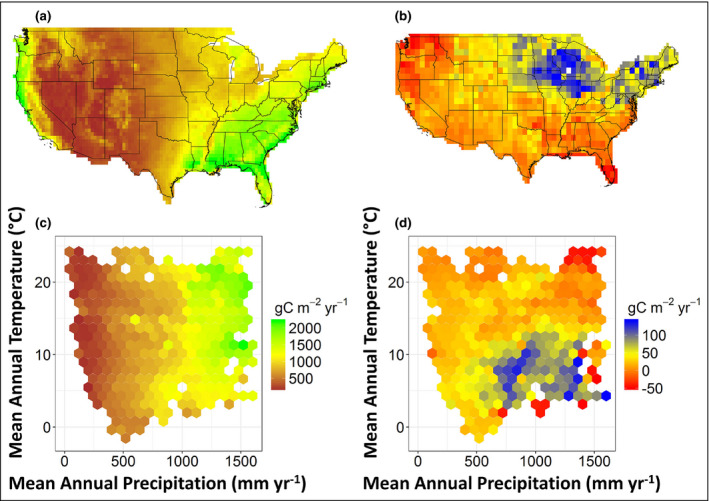
Continental‐scale estimates of mean annual GPP and net CO_2_ exchange (ie NEE) and their sensitivities to climate factors. (a) Continental‐scale estimates of GPP from MODIS satellite observations plotted within (c) their climate space of mean annual precipitation and mean annual temperature. (b) Continental‐scale estimates of NEE from atmospheric CO_2_ inversion methods plotted within (d) their climate space. All flux estimates are reported as g C/m^2^/year and have been projected to ecosystem area (modified from Liu *et al*. [2018]; see WebTable 1).

At the continental scale in the US, mean annual primary productivity and net CO_2_ exchange do not necessarily covary spatially and appear to occupy different climate spaces at regional scales (Figure 5; Liu *et al*. 2018). GPP is highest in the relatively warm and wet Southeast (Figure 5a), corresponding with high levels of mean annual precipitation (MAP, >1200 mm) across a range of mean annual temperatures (MAT, ~10–20°C; Figure 5c). In contrast, NEE is more variable, with the highest values in the Midwest (Figure 5b) at intermediate to high levels of MAP (~750–1200 mm) and lower MAT (<10°C) (Figure 5d). The spatial covariance of GPP and NEE becomes decoupled as water availability increases. We found a strong precipitation threshold of ~700 mm/year over the continental US, below which NEE is regulated by photosynthetic gains and above which NEE is regulated to a greater degree by respiration losses (Liu *et al*. 2018). This result is consistent with ecosystem‐scale studies that show the greatest response in productivity to precipitation anomalies in semi‐arid grassland and shrubland ecosystems (Knapp and Smith 2001). However, the lateral transport of C through river flow and human harvest may also be important in uncoupling GPP from NEE at continental scales. This spatial mismatch is an important finding because it is often assumed that anomalies in photosynthesis directly result in anomalies in net CO_2_ exchange. In fact, it is impossible in standard eddy covariance approaches for partitioning fluxes to have increases in net exchange without increases in photosynthesis (Reichstein *et al*. 2005), and net C exchange in land‐surface models is dominated by photosynthetic inputs (Liu *et al*. 2018).

At the global scale, the relationship between the interannual variability of the atmospheric CO_2_ growth rate and tropical land‐surface temperatures has been identified as an emergent constraint, such that higher surface temperatures diminish NEE (Cox *et al*. 2013). However, identifying the processes associated with this diminished NEE is difficult because increased tropical temperatures suppress photosynthesis and/or promote respiration, both of which lead to reduced net C exchange. It has been suggested that total respiration is the most likely mechanism explaining the emergent relationship between interannual variability in the atmospheric growth rate and tropical surface temperature (Anderegg *et al*. 2015) and that water limitation is important in regulating net CO_2_ exchange at the local scale, whereas temperature becomes more important at global scales (Jung *et al*. 2017). Recent satellite evidence suggests that terrestrial water availability that integrates temperature and precipitation variability may be the ultimate mechanism regulating interannual NEE at the global scale (Humphrey *et al*. 2018). However, evidence derived from satellite estimates of XCO_2_ and solar induced fluorescence during the recent 2015/2016 El Niño event suggest that net tropical C uptake was reduced by different processes in different tropical regions – such as reduced photosynthesis in South America, increased respiration in Africa, and increased fire emissions in Southeast Asia (Liu *et al*. 2017). Therefore, even though we are gaining new insight on the climate sensitivity of important C‐cycle processes, ecosystem‐scale observations are still lacking in certain regions to help reconcile different C‐cycle processes operating at different spatial scales.

## Conclusions and frontiers in C‐cycle research

A central goal of ecology at the macrosystem scale is to understand biosphere processes and their complex interactions with climate, land use, and changes in species distribution at regional to continental scales This has also been a central challenge of C‐cycle research because there is a long history of atmospheric CO_2_ observations that have enabled a better understanding of the C cycle at the global scale and a network of eddy covariance measurements of CO_2_ exchange at the ecosystem scale. However, reconciling differences in net CO_2_ exchange measured at these different scales continues to be difficult. We are now acquiring data from aircraft and satellites that allow important C‐cycle processes to be resolved at the biome scale. The terrestrial and aquatic ecological research communities are also compiling databases to elucidate important C‐cycle processes that may be merged to provide an integrated understanding of C transport and transformations across watersheds. Collectively, we are identifying missing pieces of the global C puzzle that now make it possible to reconcile and understand processes that help to explain discrepancies in C dynamics across scales.

## Supporting information

Fig S1

Panel S1

Table S1
