# Supplementary material for: Reconciling carbon‐cycle processes from ecosystem to global scales
Source: Front Ecol Environ. 2021 Feb 1;19(1):57–65. doi: 10.1002/fee.2296 (PMC9292898; doi:10.1002/fee.2296)
Supplement: Supplementary file 1 — Fig S1 [file FEE-19-57-s002.pdf]

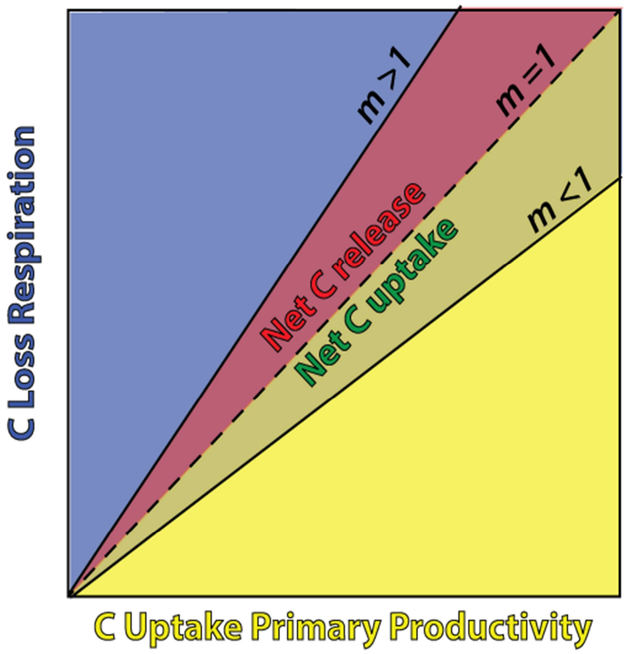

WebFigure 1. General conceptual model for net C balance across scales. When ecosystems are in steady state, $m = 1$. When photosynthetic gains exceed respiratory losses, net C uptake is observed ($m < 1$), where the integrated green area represents net C uptake. When respiratory losses exceed photosynthetic gains, net C release is observed ($m > 1$). C exchange efficiency (CEE, where CEE = net uptake/gross productivity) can therefore be approximated as $1 - m$.
