# Supplementary material for: Reconciling carbon‐cycle processes from ecosystem to global scales
Source: Front Ecol Environ. 2021 Feb 1;19(1):57–65. doi: 10.1002/fee.2296 (PMC9292898; doi:10.1002/fee.2296)
Supplement: Supplementary file 2 — Panel S1 [file FEE-19-57-s001.pdf]

AP Ballantyne *et al.* – Supporting Information

WebPanel 1. Scale-specific nomenclature of carbon dynamics

At the ***global scale***, gross global production (GGP; WebTable 1) can be approximated from satellite observations of normalized difference vegetation index (NDVI) in the terrestrial biosphere (effectively a measure of vegetation greenness) and estimates of chlorophyll concentration in marine ecosystems (Field *et al.* 1998). Based on this approach, net global production (NGP) has been estimated to be approximately 105 petagrams of carbon (C) per year (Pg C/year), with net terrestrial production (56 Pg C/year) slightly higher than net marine production (48 Pg C/year). However, several different approaches have been developed for approximating gross terrestrial production (GTP). Estimates using a light use efficiency model that assimilates satellite observations of NDVI yield relatively lower values for GTP (~110 Pg C/year; Kolby Smith *et al.* 2015) than estimates from eddy flux measurements up-scaled using satellite and climate data (GTP ~124 Pg C/year; Beer *et al.* 2010), estimates derived from the isotopic enrichment of surface waters as a result of evapotranspiration (GTP ~130 Pg C/year; Jasechko *et al.* 2013), and estimates derived from oxygen isotopes in carbon dioxide (CO_2; GTP ~150 Pg C/year; Welp *et al.* 2011). Net global exchange (NGE) is usually inferred as the difference between the amount of CO_2 emitted to the atmosphere (eg through the burning of fossil fuels and through land-use change) and the amount remaining in the atmosphere every year (ie the atmospheric CO_2 growth rate), which doubled from ~2.5 Pg C/year in 1960 to ~5.0 Pg C/year in 2010 (Ballantyne *et al.* 2012). Finally, this net global C uptake can be partitioned into net terrestrial exchange (NTE) by estimating ocean uptake independently due to air–sea gas exchange as a function of differences in partial pressure of CO_2 in the ocean with respect to the atmosphere, yielding estimates of NTE (~2.7 Pg C/year as of 2016; Le Quéré *et al.* 2016).

Total terrestrial respiration (TTR) is usually calculated as the difference between GTP and NTE (Anderegg *et al.* 2015), but is difficult to measure directly. Although trends in TTR inferred at the global scale (Ballantyne *et al.* 2017) are similar to trends estimated from soil respiration studies that have been extrapolated to the global scale (Bond-Lamberty and Thomson 2010), it is important to note that they differ in magnitude because, by definition, TTR includes aboveground autotrophic respiration that is not included in estimates of global soil respiration.

At the ***biome scale***, gross biome production (GBP; WebTable 1) can also be estimated using remotely sensed vegetation indices (eg NDVI) that are commonly assimilated into a light use efficiency model and projected over estimates of leaf area index, which allows scientists to convert measurements of absorbed photosynthetically active radiation into a currency of C flux (teragrams [Tg] C/year) at the regional to continental scale (Running *et al.* 2004). In contrast, atmospheric inversion approaches allow estimation of net terrestrial exchange (NTE) from the global to biome scale (ie net biome exchange [NBE]). Atmospheric inversions are based on the same atmospheric CO_2 mass balance principle used to estimate NGE, but often rely on land-surface models for prior CO_2 flux predictions that are then conveyed by three-dimensional atmospheric transport models using observed atmospheric circulation data. These simulated

atmospheric CO_2 concentrations are then compared to atmospheric CO_2 observations and posterior surface fluxes are updated to provide regional scale estimates of NBE (Gurney *et al.* 2002). As these atmospheric inversion approaches become more advanced in their capacity to assimilate atmospheric CO_2 measurements from atmospheric profiles (Stephens *et al.* 2007) and satellite observations of total column CO_2 (XCO_2; Houweling *et al.* 2015), they will be able to resolve NBE at increasingly finer scales (Peylin *et al.* 2013). Similarly, total biome respiration (TBR) can be inferred as the difference between GBP and NBE, but the scale of spatial autocorrelation in the errors of GBP and NBE estimates may differ, and caution should therefore be taken when trying to calculate TBR at scales smaller than continental scales, especially where data are sparse (Liu *et al.* 2018).

At the ***ecosystem scale***, a great deal has been learned about C-cycle dynamics through eddy covariance measurements (Verma *et al.* 1986). Eddy covariance measures the net ecosystem exchange of CO_2 (NEE; WebTable 1) between the Earth's surface and the atmosphere, but only intermittent measurements of respiration using sparse nighttime observations are possible. Models for total ecosystem respiration (TER) are often used to estimate gross ecosystem production (GEP), and recent studies emphasize the importance of daytime TER suppression as a critical mechanism that needs to be integrated into eddy covariance-based GEP estimates (Wehr *et al.* 2016; Keenan *et al.* 2019). There are two common complementary methods that are used to estimate these opposing fluxes: (1) extrapolate nighttime respiration to the daytime using a temperature sensitivity function to infer GEP (Reichstein *et al.* 2005), or (2) use a light use efficiency model to convert absorbed photosynthetically active radiation into the GEP C flux and then infer TER as the difference between GEP and NEE (Lasslop *et al.* 2010). It is important to note that the strong suppression of turbulence in the early evening, when soils are relatively warm, often results in a sampling bias toward colder periods at night (van Gorsel *et al.* 2007). Moreover, ecosystem respiration may be greatly reduced during daylight hours, and extrapolating nighttime values may therefore overestimate TER, and consequently GEP (Wehr *et al.* 2016; Keenan *et al.* 2019).

Freshwater aquatic ecosystems may encompass only a small proportion of the terrestrial biosphere, but streams and lakes play major roles in the transport and transformation of C from ecosystem to terrestrial biosphere scales (see Figure 1 in the main text). At the ecosystem scale, inland waters collect and process a fraction of terrestrial NEP of the catchment, and therefore this fraction of C appears to be lost from the terrestrial ecosystem. This C can then be transported downstream, where a portion of it is lost through efflux to the atmosphere or settling to sedimentation. Most aquatic ecosystems are supersaturated with respect to the partial pressure of CO_2 (pCO_2; Cole *et al.* 1994) and globally release ~2.1 Pg C/year to the atmosphere, with a larger efflux from streams (1.8 Pg C/year) than from lakes (0.3 Pg C/year) due to more efficient gas transfer in moving waters. However, these estimates are calculated from gas transfer velocities and pCO_2 measurements with respect to the atmosphere and are not direct measurements (Raymond *et al.* 2013). The second major C loss pathway in aquatic ecosystems is via sedimentation, which can be calculated by accumulation rates from sediment cores collected

from lakes and reservoirs that have been extrapolated globally to yield estimates of 0.2 Pg C/year (Cole *et al.* 2007).

While we are now able to describe and estimate major C-cycle processes from ecosystem to global scales, terminology to describe these processes has not necessarily been revised. For instance, the terms "net ecosystem exchange", "net biome production", and "net ecosystem carbon balance" are often used interchangeably to describe the difference between all C gain and all C loss pathways regardless of scale, with the assumption that more C pathways are integrated as the scale of mass balance increases (Schulze *et al.* 2000; Randerson *et al.* 2002; Chapin *et al.* 2006). We propose that a revised C-cycle nomenclature that describes consistent C-cycle processes across scales may help to alleviate confusion within and among research communities conducting C-cycle research (WebTable 1).

Conceptual model for C balance

The mass balance of CO_2 can be approximated from global to ecosystem scales, such that slight imbalances in CO_2 gain through photosynthesis and CO_2 loss through respiration are indicative of net CO_2 uptake or loss. This can be easily visualized by simply plotting CO_2 lost through respiration as a function of CO_2 gain from photosynthesis (WebFigure 1). Based on this conceptual model, and ignoring lateral transports or transformations of C, the 1:1 line represents an ecosystem in steady state (ie GEP ~ TER); ecosystems with a slope less than one (ie GEP > TER) are CO_2 sinks, and ecosystems with a slope greater than one (ie TER > GEP) are CO_2 sources. If we set the intercept to zero, this conceptual model provides a useful framework for comparing C dynamics from the ecosystem to terrestrial biosphere scale. The relative slope term (m) is unitless and can be compared across scales, such that net CO_2 exchange efficiency is equivalent to $(1 - m) \times$ GPP, with positive values representing net CO_2 uptake and negative values representing net CO_2 release to the atmosphere. This is analogous to C use efficiency (CUE), which is the amount of atmospheric C that is transferred to terrestrial biomass (where CUE = NPP/GPP). CUE is approximately 0.53 for forest ecosystems globally, but values vary considerably among forest types, with older forests and boreal forests having the lowest CUE values (DeLucia *et al.* 2007). Similarly, we can use estimates of gross primary production (GPP) and net ecosystem exchange (NEE) to estimate C exchange efficiency (CEE, where CEE = NEE/GPP or CEE = $[1 - m] \times$ GPP) as a scale-independent diagnostic metric that can be used to compare C dynamics of ecosystems within biomes or of biomes within the terrestrial biosphere (WebFigure 1).

WebReferences

Anderegg WRL, Ballantyne AP, Smith WK, *et al.* 2015. Tropical nighttime warming as a dominant driver of variability in the terrestrial carbon sink. *P Natl Acad Sci USA* **112**: 15591–96.

Ballantyne AP, Alden CB, Miller JB, *et al.* 2012. Increase in observed net carbon dioxide uptake by land and oceans during the past 50 years. *Nature* **488**: 70–72.

Ballantyne A, Smith W, Anderegg W, *et al.* 2017. Accelerating net terrestrial carbon uptake during the warming hiatus due to reduced respiration. *Nat Clim Change* **7**: 148.

Beer C, Reichstein M, Tomelleri E, *et al.* 2010. Terrestrial gross carbon dioxide uptake: global distribution and covariation with climate. *Science* **329**: 834–38.

Bond-Lamberty B and Thomson A. 2010. Temperature-associated increases in the global soil respiration record. *Nature* **464**: 579–82.

Chapin FS, Woodwell GM, Randerson JT, *et al.* 2006. Reconciling carbon-cycle concepts, terminology, and methods. *Ecosystems* **9**: 1041–50.

Cole JJ, Caraco NF, Kling GW, and Kratz TK. 1994. Carbon dioxide supersaturation in the surface waters of lakes. *Science* **265**: 1568–70.

Cole JJ, Prairie YT, Caraco NF, *et al.* 2007. Plumbing the global carbon cycle: integrating inland waters into the terrestrial carbon budget. *Ecosystems* **10**: 172–85.

DeLucia EH, Drake JE, Thomas RB, and Gonzalez-Meler M. 2007. Forest carbon use efficiency: is respiration a constant fraction of gross primary production? *Glob Change Biol* **13**: 1157–67.

Field CB, Behrenfeld MJ, Randerson JT, and Falkowski P. 1998. Primary production of the biosphere: integrating terrestrial and oceanic components. *Science* **281**: 237–40.

Gurney KR, Law RM, Denning AS, *et al.* 2002. Towards robust regional estimates of CO_2 sources and sinks using atmospheric transport models. *Nature* **415**: 626–30.

Houweling S, Baker D, Basu S, *et al.* 2015. An intercomparison of inverse models for estimating sources and sinks of CO_2 using GOSAT measurements. *J Geophys Res-Atmos* **120**: 5253–66.

Jasechko S, Sharp ZD, Gibson JJ, *et al.* 2013. Terrestrial water fluxes dominated by transpiration. *Nature* **496**: 347–50.

Keenan TF, Migliavacca M, Papale D, *et al.* 2019. Widespread inhibition of daytime ecosystem respiration. *Nature Ecol Evol* **3**: 407–15.

Kolby Smith W, Reed SC, Cleveland CC, *et al.* 2015. Large divergence of satellite and Earth system model estimates of global terrestrial CO_2 fertilization. *Nat Clim Change* **6**: 306.

Lasslop G, Reichstein M, Papale D, *et al.* 2010. Separation of net ecosystem exchange into assimilation and respiration using a light response curve approach: critical issues and global evaluation. *Glob Change Biol* **16**: 187–208.

Le Quéré C, Andrew RM, Canadell JG, *et al.* 2016. Global carbon budget 2016. *Earth Syst Sci Data* **8**: 605–49.

Liu Z, Ballantyne AP, Poulter B, *et al.* 2018. Precipitation thresholds regulate net carbon exchange at the continental scale. *Nat Commun* **9**: 3596.

Peylin P, Law RM, Gurney KR, *et al.* 2013. Global atmospheric carbon budget: results from an ensemble of atmospheric CO_2 inversions. *Biogeosciences* **10**: 6699–720.

Randerson JT, Chapin III FS, Harden JW, *et al.* 2002. Net ecosystem production: a comprehensive measure of net carbon accumulation by ecosystems. *Ecol Appl* **12**: 937–47.

Raymond PA, Hartmann J, Lauerwald R, *et al.* 2013. Global carbon dioxide emissions from inland waters. *Nature* **503**: 355–59.

Reichstein M, Falge E, Baldocchi D, *et al.* 2005. On the separation of net ecosystem exchange into assimilation and ecosystem respiration: review and improved algorithm. *Glob Change Biol* **11**: 1424–39.

Running SW, Nemani RR, Heinsch FA, *et al.* 2004. A continuous satellite-derived measure of global terrestrial primary production. *BioScience* **54**: 547–60.

Schulze E-D, Wirth C, and Heimann M. 2000. Managing forests after Kyoto. *Science* **289**: 2058–59.

Stephens BB, Gurney KR, Tans PP, *et al.* 2007. Weak northern and strong tropical land carbon uptake from vertical profiles of atmospheric CO_2. *Science* **316**: 1732–35.

van Gorsel E, Leuning R, Cleugh HA, *et al.* 2007. Nocturnal carbon efflux: reconciliation of eddy covariance and chamber measurements using an alternative to the u*-threshold filtering technique. *Tellus B* **59**: 397–403.

Verma SB, Baldocchi DD, Anderson DE, *et al.* 1986. Eddy fluxes of CO_2, water vapor, and sensible heat over a deciduous forest. *Bound-Lay Meteorol* **36**: 71–91.

Wehr R, Munger JW, McManus JB, *et al.* 2016. Seasonality of temperate forest photosynthesis and daytime respiration. *Nature* **534**: 680–83.

Welp LR, Keeling RF, Meijer HAJ, *et al.* 2011. Interannual variability in the oxygen isotopes of atmospheric CO_2 driven by El Niño. *Nature* **477**: 579–82.
