# Supplementary material for: Reconciling carbon‐cycle processes from ecosystem to global scales
Source: Front Ecol Environ. 2021 Feb 1;19(1):57–65. doi: 10.1002/fee.2296 (PMC9292898; doi:10.1002/fee.2296)
Supplement: Supplementary file 3 — Table S1 [file FEE-19-57-s003.pdf]

AP Ballantyne *et al*. – Supporting Information

WebTable 1. Important carbon-cycle processes and the scales at which they are measured

Scale (units)	Gross primary production	Net primary production	Net exchange	Total respiration
Global (Pg C/year)[a]	Gross global production (GGP)	Net global production (NGP)	Net global exchange (NGE)	Total global respiration (TGR)
Terrestrial (Pg C/year)	Gross terrestrial production (GTP)	Net terrestrial production (NTP)	Net terrestrial exchange (NTE)	Total terrestrial respiration (TTR)
Biome (Tg C/year)[b]	Gross biome production (GBP)	Net biome production (NBP)	Net biome exchange (NBE)	Total biome respiration (TBR)
Ecosystem (g C/m^2/year)	Gross ecosystem production (GEP)	Net ecosystem production (NEP)	Net ecosystem exchange (NEE)	Total ecosystem respiration (TER)

Notes: proposed revision to terminology describing net and gross carbon (C)-cycle processes at various scales. [a]Pg: petagram (equivalent of 10^{15} g of C); [b]Tg: teragram (equivalent of 10^{12} g of C).
